# Análise Comparativa do ECG com o Holter na avaliação da Frequência Cardíaca na Insuficiência Cardíaca com Fração de Ejeção Reduzida e Ritmo Sinusal

**DOI:** 10.36660/abc.20230771

**Published:** 2024-08-14

**Authors:** Fabio Eduardo Camazzola, Pedro Vellosa Schwartzmann, Marcelo Sabedotti, Rafael Massuti, Tulio Zortea, Vitoria Chen, Ana Carolina Guimarães Maggi, Francine Fonseca de Souza, Andressa da Silva Cardoso, Luciano da Silva Selistre

**Affiliations:** 1 Universidade de Caxias do Sul Caxias do Sul RS Brasil Universidade de Caxias do Sul (UCS) – Medicina, Caxias do Sul, RS – Brasil; 2 Sociedade Brasileira de Cardiologia Programa de Pós-Graduação em Insuficiência Cardíaca Rio de Janeiro RJ Brasil Sociedade Brasileira de Cardiologia – Programa de Pós-Graduação em Insuficiência Cardíaca, Rio de Janeiro, RJ – Brasil; 3 Hospital Unimed Ribeirão Preto Ribeirão Preto SP Brasil Hospital Unimed Ribeirão Preto, Ribeirão Preto, SP – Brasil; 4 Instituto de Cardiologia da Serra Caxias do Sul RS Brasil Instituto de Cardiologia da Serra, Caxias do Sul, RS – Brasil

**Keywords:** Frequência Cardíaca, Insuficiência Cardíaca, Eletrocardiografia, Eletrocardiografia Ambulatorial

## Abstract

**Fundamento:**

A frequência cardíaca (FC) na insuficiência cardíaca com fração de ejeção reduzida (ICFEr) e ritmo sinusal apresenta valor prognóstico. Entretanto, o método de mensuração é debatido na literatura.

**Objetivos:**

Comparar em pacientes com ICFEr e ritmo sinusal a FC no Holter com três eletrocardiogramas de repouso: ECG1, ECG2 e ECG3.

**Metodologia:**

Estudo transversal com 135 pacientes portadores de insuficiência cardíaca com fração de ejeção ≤ 40% e ritmo sinusal. A FC foi avaliada por ECG e Holter. Análises incluíram o coeficiente de correlação intraclasse (CCI), regressão robusta, raiz do erro quadrático médio, Bland-Altman e a área sobre a curva ROC. Adotou-se nível de significância de 0,05 e o ajuste de Bonferroni-Holm para minimizar erros tipo I.

**Resultados:**

As medianas [intervalo interquartil] de idade e fração de ejeção foram de 65 anos [16] e 30% [11], respectivamente. O CCI dos 3 ECG foi de 0,922 (intervalo de confiança de 95%: 0,892; 0,942). Os coeficientes de regressão robusta para ECG1 e ECG3 foram 0,20 (intervalo de confiança de 95%: 0,12; 0,29) e 0,21 (intervalo de confiança de 95%: 0,06; 0,36). O R^2^ robusto foi de 0,711 (intervalo de confiança de 95%: 0,628; 0,76). Na análise de concordância de Bland-Altman, os limites de concordância foram de −17,0 (intervalo de confiança de 95%: −19,0; −15,0) e 32,0 (intervalo de confiança de 95%: 30,0; 34,0). A área sob a curva ROC foi de 0,896 (intervalo de confiança de 95%: 0,865; 0,923).

**Conclusão:**

A FC do ECG mostrou alta concordância com a FC do Holter, validando seu uso clínico em pacientes com ICFEr e ritmo sinusal. Contudo, a concordância foi subótima em um terço dos pacientes com FC inferior a 70 bpm pelo ECG, devendo ser considerada a realização de Holter neste contexto.

**Figura Central:**
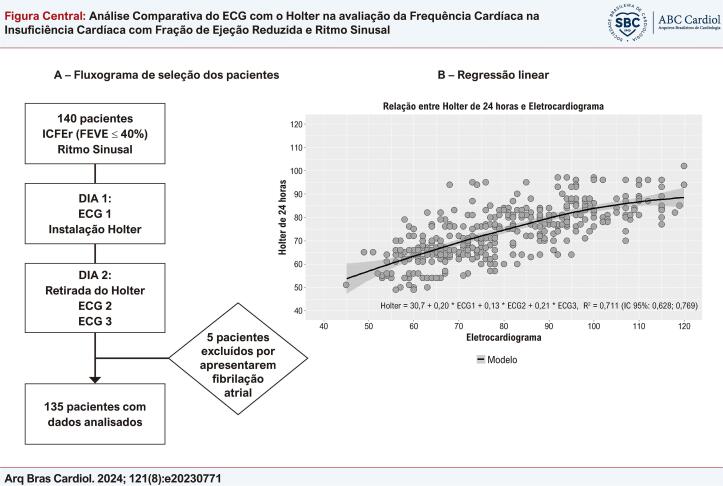
: Análise Comparativa do ECG com o Holter na avaliação da Frequência Cardíaca na Insuficiência Cardíaca com Fração de Ejeção Reduzida e Ritmo Sinusal Análise comparativa da FC no ECG versus Holter. ECG: eletrocardiograma; FC: frequência cardíaca; FEVE: fração de ejeção do ventrículo esquerdo; IC: intervalo de confiança; ICFER: insuficiência cardíaca com fração de ejeção reduzida.

## Introdução

A insuficiência cardíaca com fração de ejeção reduzida (ICFEr) segue como condição clínica com elevada morbimortalidade a despeito de avanços na terapêutica que melhoraram substancialmente os resultados nas últimas duas décadas.^1^ A frequência cardíaca (FC) de repouso é um fator de risco independente para mortalidade total e mortalidade cardiovascular na população geral e em pacientes portadores de insuficiência cardíaca.^2^ As evidências na literatura demonstram a eficácia da redução da FC nos desfechos cardiovasculares em subanálises de grandes estudos que utilizaram drogas cronotrópicas negativas. Os estudos com betabloqueadores, como o CIBIS-II (bisoprolol),^3^ MERIT-HF (succinato de metoprolol)^4^ e COMET (carvedilol e succinato de metoprolol),^5^ e estudos com a ivabradina (SHIFT e BEAUTIFUL)^6 , 7^ demonstraram a efetividade desses fármacos na prevenção do remodelamento cardíaco e no incremento de sobrevida de pacientes com insuficiência cardíaca crônica estável.^8^Esse conjunto de dados não deixa dúvida de que a FC deve ser valorizada como importante elemento prognóstico e que deve ser alvo de tratamento.^9^As diretrizes para o manejo da ICFEr crônica estável recomendam valores de FC de repouso abaixo de 70 batimentos por minuto (bpm) para melhorar os desfechos cardiovasculares em pacientes com ritmo sinusal.^1 , 10 - 12^

Existem limitações na aferição da FC dependentes do observador em cada consulta, dos diferentes períodos do dia, das diferentes circunstâncias, incluindo a possível ocorrência do fenômeno da taquicardia do avental branco e, também, do modo de aferição.^2 , 12^Como a FC em repouso no consultório pode variar de acordo com a hora do dia e a situação do registro, é útil saber se a FC medida pelo Holter 24 horas responde de forma semelhante à FC de repouso no consultório.^13^ Estudo de Pastor-Perez et al. comparou a FC de repouso com a FC média do Holter em um período de 7 dias e demonstrou que a FC de repouso parece ser adequada para estimar a FC, porém, evidenciou que a concordância foi subótima em um quarto dos pacientes quando categorizados para um alvo de FC < 70 bpm.^14^De acordo com as diretrizes correntes, os pacientes com FC de repouso < 70 bpm, porém com FC média no Holter ≥ 70 poderiam receber otimização do tratamento. O controle inadequado da FC durante monitorização prolongada, a despeito de ser encontrada FC de repouso < 70 bpm, é uma possibilidade que pode viabilizar uma oportunidade de tratamento mais intensivo com agentes redutores da FC.

A maneira mais precisa de avaliar a FC no paciente com insuficiência cardíaca ainda não está claramente definida. Na prática, a FC é avaliada durante o exame físico através da palpação do pulso, pela ausculta cardíaca ou através da realização de eletrocardiograma (ECG) de repouso, sendo esta última a estratégia adotada para a avaliação na maioria dos ensaios clínicos. Uma alternativa para a avaliação da FC por um período prolongado é o Holter. O objetivo do presente estudo é comparar a FC obtida no ECG de repouso com a FC média no Holter de 24 horas em pacientes com ICFEr e ritmo sinusal.

## Métodos

### Desenho do estudo

Estudo transversal, não intervencionista e prospectivo. O desenho do estudo seguiu as normas do STARD Statement *.*

### População do estudo

A população do estudo foi selecionada no período de 21 de setembro de 2022 a 30 de junho de 2023. Foram recrutados 140 pacientes atendidos nos ambulatórios de cardiologia do Centro Clínico da Universidade de Caxias do Sul (CECLIN/UCS), Hospital Geral de Caxias do Sul e em consultórios de cardiologistas na cidade de Caxias do Sul. Foram incluídos pacientes com idade acima de 18 anos, com diagnóstico de ICFEr, FEVE ≤ a 40%, documentada em ecocardiograma nos últimos 12 meses e que apresentavam ritmo sinusal. Os critérios de exclusão foram apresentar marcapasso, desfibrilador ou ressincronizador, estar com sinais/sintomas de insuficiência cardíaca descompensada, participação concomitante em estudo intervencional e recusa em assinar o Termo de Consentimento Livre e Esclarecido (TCLE). Foram excluídos da análise 5 pacientes por apresentarem fibrilação atrial durante os procedimentos do protocolo do estudo ( Figura Central A).

### Considerações éticas

O projeto foi submetido e aprovado pelo Comitê de Ética em Pesquisa da Universidade de Caxias do Sul sob o parecer número 5.601.769 de 24 de agosto de 2022. Todos os procedimentos envolvidos nesse estudo estão de acordo com a Declaração de Helsinki de 1975, atualizada em 2013. Todos os pacientes assinaram o TCLE.

### Procedimentos do estudo

Após a assinatura do TCLE, foi realizada a coleta dos dados demográficos, informações clínicas, resultados de exames laboratoriais e dados ecocardiográficos através de entrevista e revisão de prontuário. O exame NT-proBNP foi um parâmetro laboratorial avaliado para a maioria dos pacientes. Na indisponibilidade deste, em 9 pacientes nos quais estava disponível apenas o exame laboratorial BNP, houve conversão do mesmo para NT-proBNP com a utilização de fórmula validada por Kasahara et al.^15^ A avaliação da FC foi efetuada com ECG de 12 derivações, em posição supina após um período mínimo de 5 minutos de repouso, em 3 ocasiões por um período de 24 horas e com o Holter de 24 horas. Os exames foram realizados no Instituto de Cardiologia da Serra por equipe treinada no protocolo do estudo. No dia 1, o paciente inicialmente realizava o primeiro ECG (ECG1) e na sequência instalava-se o Holter. Após um período de 24 horas, o paciente retornava ao local para a retirada do Holter e, na sequência, a realização de 2 ECGs (ECG2 e ECG3) com intervalo de 10 minutos. Os ECGs foram realizados no aparelho Cardiete®. Os Holter foram realizados no aparelho Cardio light® da empresa Cardios® ( Figura Central A).

### Modelagem estatística

#### Cálculo amostral

A análise de dados foi realizada utilizando o software estatístico R. Consideramos um cálculo amostral para medidas repetidas através de análise multivariada da variância (MANOVA): com interações intra e entre grupos, sendo o tamanho de efeito de 20% entre as diferenças do Holter e ECG, com um erro alfa de 5% e erro beta de 80, tendo um valor crítico para estatística F de 2,64 e parâmetro λ de 11,08. O número total de pacientes calculado foi de 140, cada um com 3 medidas mínimas de ECG de repouso em períodos distintos. Para assegurar a confiabilidade e validade dos resultados, empregamos uma série de técnicas estatísticas que são descritas em detalhe abaixo.

#### Análise exploratória de dados

Inicialmente, foi realizada uma análise exploratória para sumarizar as características principais dos dados. Utilizamos como medida de tendência central a mediana e, como medida de dispersão, o intervalo interquartil (IQR) para descrever as variáveis numéricas por não possuírem distribuição normal com o teste de Shapiro-Wilk. Para as variáveis categóricas, foram calculadas frequências e porcentagens.

#### Análise de confiabilidade intraclasse

A confiabilidade intraclasse foi estimada mediante a aplicação do coeficiente de correlação intraclasse (ICC) para quantificar a concordância entre as 3 mensurações de ECG em repouso. Este coeficiente discrimina a proporção da variância total atribuível às diferenças interindividuais, distinta daquela oriunda das flutuações inerentes ao processo de medição. O modelo específico foi selecionado por sua aplicabilidade em situações em que as medições são consideradas fixas e se almeja a extrapolação dos achados para uma população mais abrangente.

Prosseguindo com a análise, a presente pesquisa contemplou a comparação entre as leituras do Holter e o ECG padrão na identificação de FC inferiores a 70 bpm. Foi desenvolvida uma variável dicotômica para designar concordância nas ocorrências em que ambas as técnicas registraram frequências abaixo do limiar estabelecido. A análise subsequente focada nos registros com frequência no Holter abaixo de 70 bpm revelou alinhamento com os dados do ECG. Empregou-se a metodologia de *bootstrapping* com 2000 interações para estimar a média e delimitar os intervalos de confiança que descrevem a proporção de verdadeiros positivos para o limiar de FC menor que 70 bpm obtido pelo Holter.

#### Regressão robusta com *bootstrapping*

A regressão robusta avaliou a relação entre a FC medida pelo Holter de 24 horas e as aferições obtidas por 3 diferentes ECGs de repouso (ECG1, ECG2, ECG3). Essa técnica estatística visa fornecer estimativas de parâmetros confiáveis e resistentes à presença de valores extremos ou a violações das suposições típicas da regressão, como a normalidade dos resíduos e a homocedasticidade. Essa análise permitiu calcular o índice de determinação robusto (R^2^ robusto) e a raiz do erro quadrático médio (RMSE, do inglês *root mean square error* ) que é uma medida de erro que quantifica a diferença média entre os valores previstos e os observados no modelo.^16^

#### Análise de concordância entre métodos de Bland-Altman

A análise de Bland-Altman representa a concordância entre o Holter de 24 horas e os ECGs. O eixo X do gráfico de Bland-Altman representou as médias entre as medidas obtidas pelo Holter de 24 horas e os ECGs, enquanto o eixo Y exibiu as diferenças entre as duas técnicas. Calculamos o viés, como a média da diferença entre o Holter e os 3 ECGs, com os limites de concordância, definidos como o viés mais ou menos 1,96 vezes o desvio padrão das diferenças. Para avaliar a incerteza associada a esses parâmetros, também foram calculados os intervalos de confiança de 95% para o viés e os limites de concordância.

#### Regressão por equações generalizadas e curva ROC

Utilizamos a regressão por equações generalizadas para modelar a relação entre os 3 ECGs e o Holter de 24 horas, com foco na capacidade de discriminação do modelo. A técnica ROC ( *receiver operating characteristic* ) foi aplicada para calcular a área sob a curva, acurácia, sensibilidade, especificidade, valor preditivo positivo, valor preditivo negativo, razão de probabilidade positiva e razão de probabilidade negativa.

#### Intervalo de confiança

A técnica de reamostragem de *bootstrapping* com 2000 replicações foi usada para estimar intervalos de confiança de 95% para todos os parâmetros.

#### Nível de significância

O nível de significância de 0,05 foi adotado em todas as análises estatísticas. Para minimizar erros tipo I decorrentes de múltiplas comparações, utilizamos o método de ajuste de Bonferroni-Holm. Este ajuste é crucial em nosso estudo, que compara diversas medidas de ECG com dados do Holter de 24 horas. Ele aumenta a confiabilidade dos resultados, reduzindo a probabilidade de falsos positivos.

O programa R versão 4.3.2 com os pacotes dplyr, boot, ggplot2, epiR, agRee e robust foi implementado às modelagens estatísticas.

## Resultados

### Estatísticas descritivas dos participantes do estudo

Tabela 1 apresenta características demográficas e clínicas com a amostra apresentando idade mediana de 65 anos [IQR: 16], 85 pacientes (63%) homens, 109 (80,7%) em classe NYHA II e III; sendo a etiologia isquêmica mais prevalente em 88 (65,2%). Em relação aos dados de exames, a fração de ejeção do ventrículo esquerdo com mediana de 30% [IQR: 11] e o valor do NT-proBNP mediano 1.345 pg/mL [IQR: 2.348,2].

**Tabela 1:** – Características Clínicas e Demográficas (n=135)

Idade, anos	65 [IQR: 16]
**Sexo masculino, n (%)**	85 (63%)
**Etnia, n (%)**	Branco – 112 (83%)
	Afrodescendente – 23 (17%)
**NYHA, n (%)**	I – 20 (14,8%)
	II – 69 (51,1%)
	III – 40 (29,6%)
	IV – 6 (4,4%)
**Fração de ejeção, %**	30% [IQR:11]
**Etiologia isquêmica, n (%)**	73 (54,1%)
**BRE, n (%)**	31 (29,8%)
**Hospitalização, 6 meses, n (%)**	66 (49,6%)
**IMC, kg/m^2^**	27,04 [IQR: 6,2]
**HAS, n (%)**	88 (65,2%)
**DM, n (%)**	50 (37%)
**Dislipidemia, n (%)**	67 (49,6%)
**Tabagismo, n (%)**	21 (26,1%)
**NT-pro-BNP, pg/mL**	1.345 [IQR: 2.348,2]
**Creatinina, mg/dL**	1,1 [IQR: 0,49]
**TFG, mL/min/1,73 m^2^**	66,2 [IQR: 33,2]
**Potássio, mEq/L**	4,5 [IQR: 0,8]
**Sódio, mEq/L**	139 [IQR: 3]
**Hemoglobina, g/dL**	13,8 [IQR: 2,6]
**Hematócrito, %**	40,9 [IQR: 7,6]
**Holter, bpm**	76 [IQR: 17]
**ECG1, bpm**	79 [IQR:28]
**ECG2, bpm**	80 [IQR: 28]
**ECG3, bpm**	81 [IQR: 30]
**Medicação utilizada**	
Sacubitril/valsartan, n (%)	47 (34,8%)
IECA, n (%)	57 (42,3%)
BRA, n (%)	17 (12,6%)
Betabloqueador, n (%)	127 (94%)
Espironolactona, n (%)	98 (72,6%)
iSGLT2, n (%)	88 (65,2%)
Ivabradina, n (%)	11 (8,1%)
Digoxina, n (%)	11 (8,1%)
Furosemida, n (%)	74 (54,8%)
Hidralazina, n (%)	7 (5,2%)
Nitrato, n (%)	10 (7,4%)

*Valores em n (%), mediana [intervalo interquartil]. BRA: bloqueador de receptor da angiotensina II; BRE: bloqueio do ramo esquerdo; DM: diabetes mellitus; ECG: eletrocardiograma; HAS: hipertensão arterial sistêmica; IECA: inibidor da enzima conversora da angiotensina; IMC: índice de massa corporal; IQR: intervalo interquartil; iSGLT2: inibidor do cotransportador de sódio e glicose do tipo 2; NYHA: New York Heart Association; TFG: taxa de filtração glomerular pela equação CKD-EPI (Chronic Kidney Disease Epidemiology Collaboration).*

Quanto ao tratamento, 121 pacientes (91,5%) estavam utilizando algum tipo de bloqueador do sistema renina angiotensina, 127 (94%) betabloqueadores, antagonista da aldosterona em 98 (72,6%) e inibidores do cotransportador de sódio e glicose 2 em 88 (65,2%).

### Regressão robusta e análise de concordância de Bland-Altman

**Figura 1:**
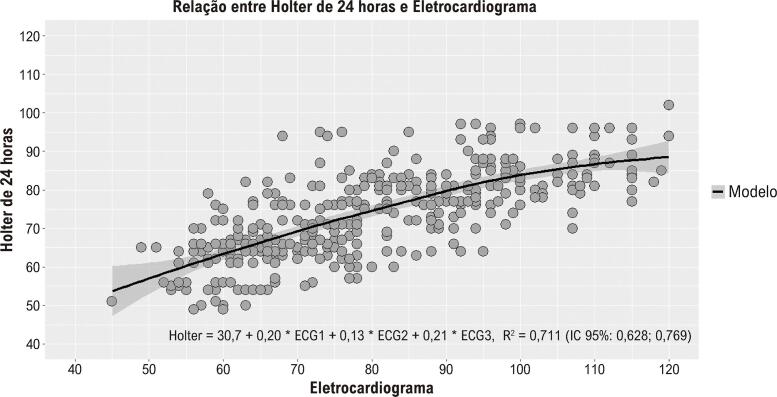
– *Regressão robusta entre eletrocardiogramas e Holter de 24 horas. ECG: eletrocardiograma; IC: intervalo de confiança.*

O coeficiente para ECG1 e ECG3 teve uma influência estatisticamente significativa no modelo, indicando uma associação positiva. Em contraste, a variável ECG2 não apresentou uma relação significativa, com seu coeficiente situando-se dentro de um intervalo de confiança que inclui valores negativos e positivos, sugerindo uma possível falta de influência sobre o modelo. A precisão do modelo foi refletida pela medida do erro padrão que indica uma variação moderada. Além disso, a correlação mostrou uma forte associação entre os métodos avaliados, com intervalo de confiança denotando uma alta certeza dessa correlação ( Figura Central B, Figura 1 e Tabela 2 ).

**Tabela 2:** – Regressão linear entre Holter e ECG

	Variável dependente
	Holter
**ECG1**	0,20 (IC 95%: 0,12; 0,29) ^‡^
**ECG2**	0,13 (IC 95%: –0,03; 0,28)
**ECG3**	0,21 (IC 95%: 0,06; 0,36) ^‡^
**Constante**	30,7 (IC 95%: 25,8; 34,5) ^‡^
**R** ^2^ **robusto**	0,711 (IC 95%: 0,628; 0,769)
**RMSE**	7,2 (IC 95%: 6,8; 7,7)
**Diferença média**	7,4 (IC 95%: 6,2; 8,7)
**Limite inferior de concordância 2,5%**	–15,0 (IC 95%: –19,0; –17,0)
**Limite superior de concordância 97,5%**	32,0 (IC 95%: 30,0; 34,0)

*ECG: eletrocardiograma; IC: intervalo de confiança; R^*2*^: índice de determinação; RMSE: raiz do erro quadrático médio. ^*‡*^p < 0,01*

**Figura 2:**
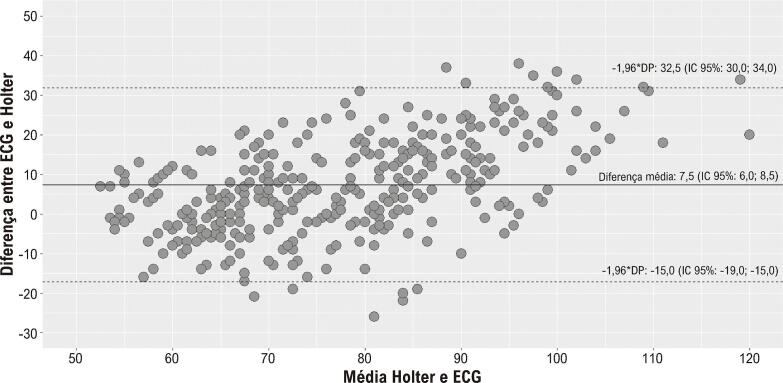
– *Gráfico de Bland-Altman. DP: desvio padrão; ECG: eletrocardiograma; IC: intervalo de confiança.*

A análise de Bland-Altman revelou uma diferença média moderada entre o Holter e os 3 ECGs, com os limites de concordância abrangendo uma ampla gama de diferenças, tanto negativas quanto positivas, indicando variações aceitáveis. Isso sugere uma concordância adequada entre os dois parâmetros, especialmente em condições de FCs mais baixas. Essa variação dentro dos limites de concordância é considerada aceitável para a prática clínica ( Tabela 2 e Figura 2 ).

### Análise de confiabilidade intraclasse

Os resultados indicaram uma concordância quase perfeita entre as medidas, conforme demonstrado por um índice de concordância, que cai dentro de uma faixa considerada de alta precisão. O intervalo de confiança estreito reforça a precisão das medidas obtidas a partir dos 3 ECGs realizados em repouso, sustentando a confiabilidade dos resultados obtidos no estudo ( Tabela 3 ).

**Tabela 3:** – Regressão por equações generalizadas e curva ROC

Parâmetros métricos	Valores
**Área sob a curva**	0,896 (IC 95%: 0,865; 0,923)
**Sensibilidade**	0,978 (IC 95%: 0,953; 1,000)
**Especificidade**	0,767 (IC 95%: 0,716; 0,817)
**Acurácia**	0,837(IC 95%: 0,836; 0,838)
**Valor preditivo positivo**	0,677 (IC 95%: 0,611; 0,743)
**Valor preditivo negativo**	0,986 (IC 95%: 0,970; 1,000)
**Razão probabilidade positiva**	4,190 (IC 95%: 3,371; 5,201)
**Razão probabilidade negativa**	0,029 (IC 95%: 0,009; 0,089)
**Coeficiente de correlação intraclasse**	0,922 (IC 95%: 0,892; 0,942)

*IC: intervalo de confiança.*

### Comparação da acurácia entre ECG e o Holter

**Figura 3:**
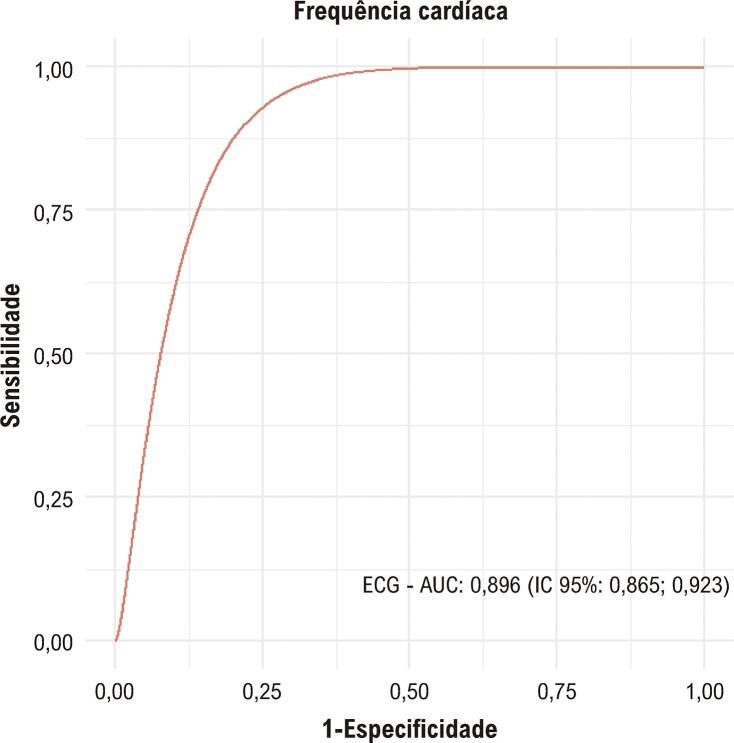
– *Curva ROC para diagnóstico de frequência cardíaca abaixo de 70 bpm com ECG. AUC: área sob a curva; ECG: eletrocardiograma; IC: intervalo de confiança.*

A curva ROC indicou uma capacidade de discriminação adequada para FCs inferiores a 70 bpm. Esta conclusão é baseada na combinação de uma elevada sensibilidade, indicando uma probabilidade significativa de detecção correta de casos positivos, com uma especificidade que demonstra uma capacidade moderada de identificar corretamente os casos negativos. Além disso, a análise revelou uma capacidade de incrementar a probabilidade de um diagnóstico relativamente preciso, evidenciada pelos valores de razão de probabilidade positiva e negativa ( Tabela 3 e Figura 3 ).

## Discussão

O controle da FC em ritmo sinusal tem adquirido uma importância cada vez maior no tratamento da ICFEr. Isso se deve a estudos que demonstraram que a FC é um forte preditor de eventos no cenário da insuficiência cardíaca. Estudos clínicos clássicos apontam para os benefícios de betabloqueadores e da ivabradina na redução de desfechos, validando a FC não apenas como um marcador de risco, mas também como um alvo terapêutico.

Diante dessas evidências, diretrizes clínicas recentes estabeleceram uma meta de FC em ritmo sinusal abaixo de 70 bpm. Nosso estudo objetivou comparar as medidas de FC obtidas através do ECG de repouso com as registradas por um monitor Holter durante um período de 24 horas em pacientes com ICFEr e ritmo sinusal. Empregamos uma variedade de métodos estatísticos, incluindo CCI, regressão linear e análises de concordância.

Os resultados demonstraram uma excelente concordância entre as medidas de FC coletadas pelo ECG de repouso e o Holter. Essa consistência foi confirmada através de várias análises estatísticas, incluindo a curva ROC, com um excelente resultado documentado pela área sob a curva.

No estudo SHIFT Holter, foram avaliados 602 pacientes com a mensuração da FC realizada no ECG de repouso e com a monitorização prolongada do Holter de 24 horas para avaliar a resposta da FC à ação da ivabradina além da aferição de repouso, observando sua reposta à monitorização prolongada no período diurno, noturno e na média das 24 horas.^13^ A reposta encontrada foi semelhante na avaliação de repouso e nos períodos diurno e noturno da monitorização prolongada. Com relação aos valores de FC encontrados, observou-se que a FC média do ECG de repouso basal foi semelhante à média diurna do Holter, contudo, foi 9 a 10 bpm superior em comparação à FC média noturna.^13^ A FC basal do consultório é inferior à média do Holter de 24 horas e à FC noturna, mas semelhante à FC diurna. A FC média de consultório no estudo foi de 78,4 ± 8,3 bpm no grupo ivabradina e 77,7 ± 8 bpm no placebo e a FC média do Holter de 24 horas foi 75,4 ± 10,3 no grupo ivabradina e 78,4 ± 9,7 no grupo placebo,^13^ não demonstrando uma diferença significativa, corroborando a validade dos achados de nosso estudo. Esses achados sugerem que as medidas da FC de repouso e da FC média do Holter de 24 horas são adequadas para a seleção de pacientes elegíveis para tratamento com agentes redutores da FC e para avaliar os efeitos do tratamento na redução da FC.

Em 2013, Pastor-Pérez et al., em estudo com 75 pacientes, compararam a FC de repouso com a FC média da monitorização prolongada no Holter durante um período de 7 dias. Os resultados encontrados em valores absolutos de FC foram similares ao nosso estudo, porém, quando os pacientes foram categorizados para FC < 70 ou ≥ 70 bpm, houve discordância em aproximadamente 25% dos casos, que apresentavam FC de repouso < 70 bpm e FC média em monitorização prolongada obtida pelo Holter ≥ 70 bpm.^14^ Em nosso estudo, com um número maior de pacientes, encontramos resultados similares; realizamos análise de *bootstrapping* não-paramétrico categorizada por FC e encontramos uma concordância de 63,8% (intervalo de confiança de 95%: 55,3; 71,6) para a FC < 70 bpm. Aproximadamente um terço dos pacientes com FC < 70 bpm no ECG de repouso apresentam FC média discordante, ou seja, acima de 70 bpm na monitorização prolongada no Holter. Esses pacientes seriam potenciais candidatos para intensificação do tratamento com agentes redutores da FC. A utilização da monitorização prolongada por meio do Holter de 24 horas deve ser considerada nos pacientes com FC de repouso < 70 bpm.

Em 2010, Bohm et al., realizou uma análise dos desfechos cardiovasculares do estudo SHIFT nos grupos placebo (n = 3.264) e ivabradina (n = 3.241), divididos por quintis da FC basal no grupo placebo. O risco de eventos do desfecho composto primário (morte cardiovascular e hospitalização por insuficiência cardíaca) aumentou 3% com o incremento de 1 batimento em relação à FC basal e 16% para cada aumento de 5 bpm.^17^ Em nosso estudo observamos uma diferença média de 7,0 (intervalo de confiança de 95%: 6,0; 8,0) do ECG em relação ao Holter 24 de horas, obtida pelo cálculo do erro padrão dos resíduos. A diferença encontrada se justifica principalmente pela redução fisiológica da FC no período sono que influencia na média do Holter e que já foi demonstrada no estudo SHIFT Holter.^13^A impressão é que não há relevância clínica por essa diferença. Uma resposta definitiva a esse questionamento seria trazida por ensaio clínico no qual o uso das medicações redutoras da FC fosse guiado pelos valores da FC encontrados no Holter.

Os pacientes analisados neste estudo transversal estavam otimizados do ponto de vista terapêutico; a maioria estava recebendo os medicamentos indicados nas diretrizes para o tratamento da ICFEr. Os dados mostraram também que esses resultados, quanto à qualidade do tratamento, foram melhores do que os usualmente descritos nos registros e mesmo em alguns ensaios clínicos.^1 , 9 , 11 , 18^ Com relação aos resultados do tratamento para obtenção da meta de FC < 70 bpm, observamos que a FC média registrada durante os 3 exames de ECG foi de aproximadamente 82 bpm, com medianas de 79, 80 e 81 bpm para ECG1, ECG2 e ECG 3, respectivamente. A FC média do Holter de 24 horas foi 76 bpm [IQR = 17]. Esses achados indicam que o tratamento com drogas redutoras da FC precisa ser ajustado; 94% dos pacientes faziam uso de betabloqueador, sendo que o mais utilizado foi o succinato de metoprolol com dose média de 50 mg [IQR: 50], seguido pelo carvedilol com dose média de 25 mg [IQR: 37,5] e o bisoprolol com dose média de 5 mg [IQR: 2,5]. As doses médias utilizadas são consideradas baixas, indicando uma necessidade de otimização da dose das medicações. Outra droga com efeito cronotrópico negativo, a ivabradina, foi utilizada em apenas 11 pacientes (8,1%), sendo, portanto, subutilizada como adjuvante ao betabloqueador no controle da FC dos pacientes avaliados. Estudos anteriores demonstraram que aproximadamente um terço dos pacientes com ICFEr crônica tratados em centros de referência de insuficiência cardíaca e recebendo terapia médica otimizada permaneciam com FC de repouso ≥ 70 bpm.^8 , 18^ A titulação inadequada da dose de betabloqueador e o subuso de ivabradina seriam potenciais barreiras para que os pacientes obtenham os benefícios comprovados do controle da FC.^19^

Esse é o primeiro estudo, dentro do nosso conhecimento, que trouxe dados objetivos de comparação entre a FC obtida no ECG e Holter no cenário brasileiro, dentro do contexto da ICFEr. O delineamento prospectivo com amostra probabilística permite um acompanhamento mais preciso e controlado dos participantes e o rigor estatístico ao empregar uma variedade de métodos estatísticos avançados, como MANOVA, ICC, *bootstrapping* e análise de concordância de Bland-Altman.

O estudo apresenta várias limitações, como o caráter unicêntrico, achado restrito a população de ICFEr, a ausência de avaliação de disautonomia em diabéticos e ausência de pacientes chagásicos, o que pode limitar a generalização dos resultados para populações mais diversas. O caráter transversal do estudo impede a avaliação longitudinal dos pacientes e, portanto, a análise de desfechos.

## Conclusão

Em nosso estudo, a avaliação da FC com ECG de repouso em pacientes com ICFEr e ritmo sinusal demonstrou ser um método preciso e confiável, corroborando o seu uso generalizado na prática clínica. Contudo, a concordância foi subótima em um terço dos pacientes com FC inferior a 70 bpm pelo ECG, devendo ser considerada a realização de Holter 24 de horas nessa situação.
