# Supplementary material for: Análise Comparativa do ECG com o Holter na avaliação da Frequência Cardíaca na Insuficiência Cardíaca com Fração de Ejeção Reduzida e Ritmo Sinusal
Source: Arq Bras Cardiol. 2024 Aug 14;121(8):e20230771. [Article in Portuguese] doi: 10.36660/abc.20230771 (PMC12092043; doi:10.36660/abc.20230771)
Supplement: Translation

# Translation: Comparative Analysis of ECG and Holter Monitoring in the Assessment of Heart Rate in Heart Failure with Reduced Ejection Fraction and Sinus Rhythm

**DOI:** 10.36660/abc.20230771i

**Authors:** Fabio Eduardo Camazzola, Pedro Vellosa Schwartzmann, Marcelo Sabedotti, Rafael Massuti, Tulio Zortea, Vitoria Chen, Ana Carolina Guimarães Maggi, Francine Fonseca de Souza, Andressa da Silva Cardoso, Luciano da Silva Selistre

**Affiliations:** 1 Brazil Universidade de Caxias do Sul (UCS) – Medicina, Caxias do Sul, RS – Brazil; 2 Brazil Sociedade Brasileira de Cardiologia – Programa de Pós-Graduação em Insuficiência Cardíaca, Rio de Janeiro, RJ – Brazil; 3 Brazil Hospital Unimed Ribeirão Preto, Ribeirão Preto, SP – Brazil; 4 Brazil Instituto de Cardiologia da Serra, Caxias do Sul, RS – Brazil

**Keywords:** Heart Rate, Heart Failure, Electrocardiography, Ambulatory Electrocardiography

## Abstract

**Background:**

Heart rate (HR) has shown prognostic value in patients with heart failure with reduced ejection fraction (HFrEF) and sinus rhythm. However, the method of measurement is debated in the literature.

**Objectives:**

To compare HR on Holter with 3 resting electrocardiograms (ECG1, ECG2, and ECG3) in patients with HFrEF and sinus rhythm.

**Methods:**

This was a cross-sectional study with 135 patients with heart failure with ejection fraction ≤ 40% and sinus rhythm. HR was assessed by ECG and Holter. Analyses included intraclass correlation coefficient (ICC), robust regression, root mean squared error, Bland-Altman, and area under the receiver operating characteristic (ROC) curve. A significance level of 0.05 and Bonferroni-Holm adjustment were adopted to minimize type I errors.

**Results:**

The median [interquartile range] age and ejection fraction were 65 years [16] and 30% [11], respectively. The ICC of the 3 ECGs was 0.922 (95% confidence interval: 0.892; 0.942). The robust regression coefficients for ECG1 and ECG3 were 0.20 (95% confidence interval: 0.12; 0.29) and 0.21 (95% confidence interval: 0.06; 0.36). The robust R^2^was 0.711 (95% confidence interval: 0.628; 0.76). In the Bland-Altman agreement analysis, the limits of agreement were −17.0 (95% confidence interval: −19.0; −15.0) and 32.0 (95% confidence interval: 30.0; 34.0). The area under the ROC curve was 0.896 (95% confidence interval: 0.865; 0.923).

**Conclusion:**

The HR on ECG showed high agreement with the HR on Holter, validating its clinical use in patients with HFrEF and sinus rhythm. However, agreement was suboptimal in one third of patients with HR below 70 bpm on ECG; thus, 24-hour Holter monitoring should be considered in this context.

**Central Illustration:** : Comparative Analysis of ECG and Holter Monitoring in the Assessment of Heart Rate in Heart Failure with Reduced Ejection Fraction and Sinus Rhythm Comparative analysis of heart rate on ECG versus Holter. CI: confidence interval; ECG: electrocardiogram; HFrEF: heart failure with reduced ejection fraction; LVEF: left ventricular ejection fraction.

## Introduction

Heart failure with reduced ejection fraction (HFrEF) is a clinical condition that continues to have elevated morbidity and mortality, despite therapeutic advances that have substantially improved results in the past 2 decades.^1^Resting heart rate (HR) is an independent risk factor for total mortality and cardiovascular mortality in the general population and in patients with heart failure.^2^Evidence in the literature has demonstrated the effectiveness of reducing HR in relation to cardiovascular outcomes in subanalyses of large studies that used negative chronotropic drugs. Studies with beta-blockers, such as CIBIS-II (bisoprolol),^3^MERIT-HF (metoprolol succinate),^4^and COMET (carvedilol and metoprolol succinate),^5^and studies with ivabradine (SHIFT and BEAUTIFUL)^6 , 7^have demonstrated the effectiveness of these drugs in preventing cardiac remodeling and increasing the survival of patients with stable chronic heart failure.^8^These data leave no doubt that HR should be appreciated as an important prognostic element and should be the target of treatment.^9^Guidelines for the management of stable chronic HFrEF recommend resting HR values below 70 beats per minute (bpm) to improve cardiovascular outcomes in patients with sinus rhythm.^1 , 10 - 12^

There are limitations to measuring HR depending on the observer at each visit, different times of the day, and different circumstances, including the possible occurrence of white-coat tachycardia, as well as the method of measurement.^2 , 12^As resting HR at an office can vary according to the time of day and recording situation, it is useful to know whether HR measured by 24-hour Holter monitoring corresponds to resting HR obtained at the office.^13^A study by Pastor-Perez et al. compared resting HR with mean HR on Holter over a period of 7 days and demonstrated that resting HR appeared to be adequate for estimating HR; however, their study showed that agreement was suboptimal in a quarter of patients when categorized for target HR < 70 bpm.^14^According to current guidelines, patients with resting HR < 70 bpm, but with mean Holter HR ≥ 70 should receive treatment optimization. Inadequate HR control during prolonged monitoring, in spite of the finding of resting HR < 70 bpm, may provide an opportunity for more intensive treatment with HR-lowering agents.

The most accurate way to assess HR in patients with heart failure has not yet been clearly defined. In practice, HR is assessed during physical examination by pulse palpation, cardiac auscultation, or by performing a resting electrocardiogram (ECG); the latter is the strategy adopted for assessment in most clinical trials. An alternative for assessing HR over a prolonged period is Holter monitoring. The objective of the present study was to compare HR obtained on resting ECG with mean HR on 24-hour Holter monitoring in patients with HFrEF and sinus rhythm.

## Methods

### Study design

This was a cross-sectional, non-interventional, prospective study. The study design followed the STARD Statement.

### Study population

The study population was selected from September 21, 2022 to June 30, 2023, recruiting 140 patients treated at cardiology outpatient clinics of the Clinical Center of the University of Caxias do Sul (CECLIN/UCS), Caxias do Sul General Hospital, and cardiologist offices in the city of Caxias do Sul. The study included patients over the age of 18, with a diagnosis of HFrEF, lower than or equal to 40%, documented on an echocardiogram within the last 12 months, and sinus rhythm. The exclusion criteria were as follows: having a pacemaker, defibrillator, or resynchronizer; showing signs/symptoms of decompensated heart failure; concomitant participation in an interventional study; and refusal to sign the informed consent form. Five patients were excluded from the analysis because they had atrial fibrillation during the study protocol procedures ( Central Illustration A).

### Ethical considerations

The project was submitted and approved by the Research Ethics Committee of the University of Caxias do Sul under opinion number 5.601.769 on August 24, 2022. All study procedures are in accordance with the 1975 Declaration of Helsinki (updated in 2013), and all patients signed an informed consent form.

### Study procedures

After the informed consent form was signed, demographic data, clinical information, laboratory test results, and echocardiographic data were collected by means of interviews and review of medical records. NT-proBNP was a laboratory parameter evaluated for most patients. When it was unavailable, in 9 patients whose BNP was the only laboratory exam available, it was converted to NT-proBNP using the formula validated by Kasahara et al.^15^HR was assessed using a 12-lead ECG, in supine position, after a minimum period of 5 minutes of rest, on 3 occasions, over a period of 24 hours and with 24-hour Holter. The exams were carried out at the Instituto de Cardiologia da Serra by a team trained in the study protocol. On day 1, patients initially underwent the first ECG (ECG1), and the Holter monitor was subsequently placed. After a period of 24 hours, the patients returned to the site to have the Holter removed and, subsequently, 2 ECGs (ECG2 and ECG3) were performed at 10-minute intervals. ECGs were performed using a Cardiete® device. Holter measurements were performed using a Cardio light® device made by the company Cardios® ( Central Illustration A).

### Statistical modeling

#### Sample calculation

Data analysis was performed using R statistical software. We considered a sample calculation for repeated measures through multivariate analysis of variance (MANOVA): with intra- and inter-group interactions, effect size of 20% for differences between Holter and ECG, alpha error of 5% and beta error of 80, a critical value for F statistics of 2.64, and λ parameter of 11.08. The total number of patients calculated was 140, each with at least 3 resting ECG measurements at different periods. To ensure the reliability and validity of the results, we employed a series of statistical techniques described in detail below.

#### Exploratory data analysis

Initially, exploratory analysis was carried out to summarize the main data characteristics. We used median as a measure of central tendency and interquartile range (IQR) as a measure of dispersion to describe numerical variables, as they did not show normal distribution according to the Shapiro-Wilk test. For categorical variables, frequencies and percentages were calculated.

#### Intraclass reliability analysis

Intraclass reliability was estimated by applying the intraclass correlation coefficient (ICC) to quantify the agreement between the 3 resting ECG measurements. This coefficient discriminates the proportion of total variance attributable to inter-individual differences, as distinct from that arising from fluctuations inherent to the measurement process. The specific model was selected due to its applicability in situations where measurements are considered fixed, and the aim is to extrapolate the findings to a broader population.

Continuing the analysis, this study included comparison between Holter readings and standard ECG in identifying HR below 70 bpm. A dichotomous variable was developed to designate agreement in occurrences where both techniques recorded HR below the established threshold. Subsequent analysis focused on recordings with HR on Holter below 70 bpm revealed that they were aligned with ECG data. We used the method of bootstrapping with 2000 iterations to estimate the mean and delimit the confidence intervals that described the proportion of true positives for the HR threshold lower than 70 bpm obtained by Holter.

#### Robust regression with bootstrapping

Robust regression evaluated the relationship between HR measured by 24-hour Holter and measurements obtained by 3 different resting ECGs (ECG1, ECG2, and ECG3). This statistical technique aims to provide parameter estimates that are reliable and resistant to the presence of extreme values or violations of typical regression assumptions, such as normality of residuals and homoscedasticity. This analysis made it possible to calculate the robust coefficient of determination (robust R^2^) and the root mean squared error (RMSE), which is a measurement of error that quantifies the mean difference between the predicted values and those observed in the model.^16^

#### Bland-Altman analysis of agreement between methods

Bland-Altman analysis represents the agreement between 24-hour Holter monitoring and ECGs. The X axis of the Bland-Altman graph represented the means between the measurements obtained on 24-hour Holter and the ECGs, while the Y axis displayed the differences between both techniques. We calculated the bias as the mean difference between the Holter and the 3 ECGs, with the limits of agreement, defined as the bias plus or minus 1.96 times the standard deviation of the differences. To assess the uncertainty associated with these parameters, we also calculated 95% confidence intervals for the bias and limits of agreement.

#### Regression using generalized estimating equations and ROC curve

We conducted regression using generalized estimating equations to model the relationship between the 3 ECGs and 24-hour Holter monitoring, focusing on the model’s discrimination capacity. A receiver operating characteristic (ROC) curve was applied to calculate the area under the curve, accuracy, sensitivity, specificity, positive predictive value, negative predictive value, positive likelihood ratio, and negative likelihood ratio.

#### Confidence interval

The resampling technique of bootstrapping with 2000 replications was used to estimate 95% confidence intervals for all parameters.

#### Significance level

A significance level of 0.05 was adopted in all statistical analyses. To minimize type I errors resulting from multiple comparisons, we used the Bonferroni-Holm adjustment method. This adjustment was crucial in our study, which compared multiple ECG measurements with data from 24-hour Holter monitoring, because it increased the reliability of results by reducing the likelihood of false positives.

We used R program, version 4.3.2, with the packages dplyr, boot, ggplot2, epiR, agRee, and robust for statistical modeling.

## Results

### Descriptive statistics of study participants

Table 1 displays demographic and clinical characteristics. The sample had a median age of 65 years [IQR: 16], 85 male patients (63%), 109 (80.7%) in New York Heart Association functional class II and III. Ischemic etiology was the most prevalent in 88 (65.2%). Regarding exam data, median left ventricular ejection fraction was 30% [IQR: 11], and the median NT-proBNP value was 1345 pg/mL [IQR: 2348.2].

**Table 1:** – Clinical and demographic characteristics (n=135)

Age, years	65 [IQR: 16]
**Male sex, n (%)**	85 (63%)
**Race, n (%)**	White – 112 (83%)
	Black – 23 (17%)
**NYHA functional class, n (%)**	I – 20 (14.8%)
	II – 69 (51.1%)
	III – 40 (29.6%)
	IV – 6 (4.4%)
**Ejection fraction, %**	30% [IQR:11]
**Ischemic etiology, n (%)**	73 (54.1%)
**LBBB, n (%)**	31 (29.8%)
**Hospitalization, 6 months, n (%)**	66 (49.6%)
**BMI, Kg/m^2^**	27.04 [IQR: 6.2]
**SAH, n (%)**	88 (65.2%)
**DM, n (%)**	50 (37%)
**Dyslipidemia, n (%)**	67 (49.6%)
**Tobacco use, n (%)**	21 (26.1%)
**NT-pro-BNP, pg/mL**	1.345 [IQR: 2.348.2]
**Creatinine, mg/dL**	1.1 [IQR: 0.49]
**GFR, mL/min/1,73 m^2^**	66.2 [IQR: 33.2]
**Potassium, mEq/L**	4.5 [IQR: 0.8]
**Sodium, mEq/L**	139 [IQR: 3]
**Hemoglobin, g/dL**	13.8 [IQR: 2.6]
**Hematocrit, %**	40.9 [IQR: 7.6]
**Holter, bpm**	76 [IQR: 17]
**ECG1, bpm**	79 [IQR:28]
**ECG2, bpm**	80 [IQR: 28]
**ECG3, bpm**	81 [IQR: 30]
**Medication use**	
Sacubitril/valsartan, n (%)	47 (34.8%)
ACEI, n (%)	57 (42.3%)
ARB, n (%)	17 (12.6%)
Beta-blocker, n (%)	127 (94%)
Spironolactone, n (%)	98 (72.6%)
SGLT2i, n (%)	88 (65.2%)
Ivabradine, n (%)	11 (8.1%)
Digoxin, n (%)	11 (8.1%)
Furosemide, n (%)	74 (54.8%)
Hydralazine, n (%)	7 (5.2%)
Nitrate, n (%)	10 (7.4%)

*Values shown as n (%) and median [interquartile range]. ACEI: angiotensin-converting enzyme inhibitor; ARB: angiotensin II receptor blocker; BMI: body mass index; DM: diabetes mellitus; ECG: electrocardiogram; GFR: glomerular filtration rate calculated using the CKD-EPI (Chronic Kidney Disease Epidemiology Collaboration) equation; IQR: interquartile range; LBBB: left bundle branch block; NYHA: New York Heart Association; SAH: systemic arterial hypertension; SGLT2i: sodium glucose cotransporter 2 inhibitor.*

Regarding treatment, 121 patients (91.5%) were using some type of renin-angiotensin system blocker; 127 (94%) were using beta-blockers; 98 (72.6%) were using an aldosterone antagonist; and 88 (65.2%) were using sodium-glucose cotransporter 2 inhibitors.

### Robust regression and Bland-Altman agreement analysis

The coefficient for ECG1 and ECG3 had a statistically significant influence on the model, indicating a positive association. In contrast, the variable ECG2 did not show a significant relationship; its coefficient was situated within a confidence interval that included negative and positive values, suggesting a possible lack of influence on the model. The accuracy of the model was reflected by the standard error measure, which indicated moderate variation. Furthermore, the correlation showed a strong association between the methods evaluated, with a confidence interval denoting a high certainty for this correlation ( Central Illustration B, Figure 1 and Table 2 ).

**Figure 1:** – *Robust regression between electrocardiogram and 24-hour Holter. CI: confidence interval; ECG: electrocardiogram.*

**Table 2:** – Linear regression between Holter and ECG

	Dependent variable
	Holter
**ECG1**	0.20 (95% CI: 0.12; 0.29)^‡^
**ECG2**	0.13 (95% CI: –0.03; 0.28)
**ECG3**	0.21 (95% CI: 0.06; 0.36)^‡^
**Constant**	30.7 (95% CI: 25.8; 34.5)^‡^
**Robust R^**2**^**	0.711 (95% CI: 0.628; 0.769)
**RMSE**	7.2 (95% CI: 6.8; 7.7)
**Mean difference**	7.4 (95% CI: 6.2; 8.7)
**Lower limit of agreement, 2.5%**	–15.0 (95% CI: –19.0; –17.0)
**Upper limit of agreement, 97.5%**	32.0 (95% CI: 30.0; 34.0)

*CI: confidence interval; ECG: electrocardiogram; R2: coefficient of determination; RMSE: root mean squared error.^*‡*^p < 0.01*

Bland-Altman analysis revealed a moderate mean difference between the Holter and the 3 ECGs, with the limits of agreement encompassing a wide range of differences, both negative and positive, indicating acceptable variations. This suggests adequate agreement between both parameters, especially under conditions of lower HR. This variation within the limits of agreement is considered acceptable for clinical practice ( Table 2 and Figure 2 ).

**Figure 2:** – *Bland-Altman graph. CI: confidence interval; ECG: electrocardiogram; SD: standard deviation.*

### Intraclass reliability analysis

The results indicated almost perfect agreement between the measurements, as demonstrated by the agreement index, which fell within a range considered highly accurate. The narrow confidence interval reinforces the accuracy of the measurements obtained from the 3 resting ECGs performed, supporting the reliability of the results obtained in the study ( Table 3 ).

**Table 3:** – Regression using generalized estimating equations and receiver operating characteristic curve

Metric parameters	Values
**Area under the curve**	0.896 (95% CI: 0.865; 0.923)
**Sensitivity**	0.978 (95% CI: 0.953; 1.000)
**Specificity**	0.767 (95% CI: 0.716; 0.817)
**Accuracy**	0.837(95% CI: 0.836; 0.838)
**Positive predictive value**	0.677 (95% CI: 0.611; 0.743)
**Negative predictive value**	0.986 (95% CI: 0.970; 1.000)
**Positive likelihood ratio**	4.190 (95% CI: 3.371; 5.201)
**Negative likelihood ratio**	0.029 (95% CI: 0.009; 0.089)
**Intraclass correlation coefficient**	0.922 (95% CI: 0.892; 0.942)

*CI: confidence interval.*

### Comparison of accuracy between ECG and Holter

The ROC curve indicated adequate discrimination capacity for HR below 70 bpm. This conclusion is based on the combination of a high sensitivity, indicating a significant probability of correctly detecting positive cases, with a specificity that demonstrates a moderate ability to correctly identify negative cases. Furthermore, analysis revealed an ability to increase the probability of a relatively accurate diagnosis, as shown by the positive and negative likelihood ratio values ( Table 3 and Figure 3 ).

**Figure 3:** – *Receiver operating characteristic curve for diagnosis of heart rate below 70 bpm with ECG. AUC: area under the curve; CI: confidence interval; ECG: electrocardiogram.*

## Discussion

It has become increasingly important to control HR in sinus rhythm when treating HFrEF. This is owing to studies that have demonstrated that HR is a strong predictor of events in the setting of heart failure. Classic clinical studies have indicated the benefits of beta-blockers and ivabradine in reducing outcomes, validating HR not only as a risk marker, but also a therapeutic target.

In view of this evidence, recent clinical guidelines have established a goal of HR in sinus rhythm below 70 bpm. The objective of our study was to compare HR measurements obtained through resting ECG with those recorded by a Holter monitor over a 24-hour period in patients with HFrEF and sinus rhythm. We employed a variety of statistical methods, including ICC, linear regression, and agreement analyses.

The results demonstrated excellent agreement between HR measurements obtained by resting ECG and Holter. This consistency was confirmed through several statistical analyses, including ROC curve, with an excellent result according to the area under the curve.

The SHIFT Holter study evaluated 602 patients with HR measured on resting ECG and 24-hour Holter monitoring to evaluate the HR response to the action of ivabradine, in addition to resting measurement, observing their response to monitoring prolonged during the daytime, nighttime, and 24-hour mean.^13^The response found was similar in the resting assessment to the daytime and nighttime periods of prolonged monitoring. In relation to the HR values found, the mean HR of the baseline resting ECG was similar to the daytime mean of the Holter; however, it was 9 to 10 bpm higher compared to the mean nocturnal HR.^13^The baseline HR at the office is lower than the mean 24-hour Holter and nocturnal HR, but similar to daytime HR. In their study, the mean HR at the office was 78.4 ± 8.3 bpm in the ivabradine group and 77.7 ± 8 bpm in the placebo group, and the mean 24-hour Holter HR was 75.4 ± 10.3 in the ivabradine group and 78.4 ± 9.7 in the placebo group,^13^not showing a significant difference, corroborating the validity of the findings of our study. These findings suggest that resting HR measurements and mean HR on 24-hour Holter are appropriate for selecting patients who are eligible for treatment with HR-lowering agents, as well as for assessing the HR-lowering effects of treatment.

Pastor-Pérez et al., in a 2013 study with 75 patients, compared resting HR with the mean HR from prolonged Holter monitoring over a period of 7 days. The results found in absolute HR values were similar to our study; however, when patients were categorized as HR < 70 or ≥ 70 bpm, there was disagreement in approximately 25% of cases, which showed resting HR < 70 bpm and HR ≥ 70 bpm on prolonged Holter monitoring.^14^In our study, with a larger number of patients, we found similar results. We performed non-parametric bootstrapping analysis categorized by HR and found an agreement of 63.8% (95% confidence interval: 55.3; 71.6) for HR < 70 bpm. Approximately one third of patients with HR < 70 bpm on resting ECG had a discordant mean HR, that is, above 70 bpm on prolonged Holter monitoring. These patients would be potential candidates for intensification of treatment with HR-lowering agents. The use of prolonged 24-hour Holter monitoring should be considered in patients with resting HR < 70 bpm.

In 2010, Bohm et al., carried out an analysis of the cardiovascular outcomes of the SHIFT study in the placebo (n = 3,264) and ivabradine (n = 3,241) groups, divided by quintiles of baseline HR in the placebo group. The risk of events in the primary composite endpoint (cardiovascular death and hospitalization for heart failure) increased by 3% with every 1 beat increase in relation to the baseline HR and 16% for each 5-bpm increase.^17^In our study we observed a mean difference of 7.0 (95% confidence interval: 6.0; 8.0) between ECG and 24-hour Holter, obtained by calculating the standard error of the residuals. The difference found is mainly justified by the physiological reduction in HR during the sleep period, which influences the mean Holter, as previously demonstrated in the SHIFT Holter study.^13^The impression is that there is no clinical relevance for this difference. A definitive answer to this question would be provided by a clinical trial in which the use of HR-reducing medications was guided by the HR values found on Holter monitoring.

The patients analyzed in this cross-sectional study were optimized from a therapeutic point of view; the majority were receiving the medications indicated in the guidelines for the treatment of HFrEF. The data also showed that these results, regarding the quality of treatment, were better than those usually described in records and even in some clinical trials.^1 , 9 , 11 , 18^In relation to the results of treatment to achieve the HR goal < 70 bpm, we observed that the mean HR recorded during the 3 ECGs was approximately 82 bpm, with medians of 79, 80, and 81 bpm for ECG1, ECG2, and ECG 3, respectively. The median HR on 24-hour Holter was 76 bpm [IQR = 17]. These findings indicate that treatment with HR-lowering drugs needs to be adjusted; 94% of patients were using beta-blockers. The most used was metoprolol succinate with a median dose of 50 mg [IQR: 50], followed by carvedilol with a median dose of 25 mg [IQR: 37.5], and bisoprolol with a median dose of 5 mg [IQR: 2.5]. The mean doses used are considered low, indicating a need to optimize the medication dosage. Another drug with a negative chronotropic effect, ivabradine, was used in only 11 patients (8.1%), and was thus underused as an adjuvant to beta-blockers to control HR in the patients evaluated. Previous studies have demonstrated that approximately one third of patients with chronic HFrEF treated at heart failure referral centers receiving optimized medical therapy maintained resting HR ≥ 70 bpm.^8 , 18^Inadequate beta-blocker dose titration and underuse of ivabradine would be potential barriers to patients reaching the proven benefits of HR control.^19^

To the best of our knowledge, this was the first study to provide objective data comparing HR obtained from ECG and Holter monitoring in Brazil, within the context of HFrEF. The prospective design with probabilistic sampling allowed for more accurate and controlled participant monitoring, in addition to statistical rigor employing a variety of advanced statistical methods, such as MANOVA, ICC, bootstrapping, and Bland-Altman agreement analysis.

This study has several limitations, for example, its single-center design, findings restricted to the population with HFrEF, the lack of assessment of dysautonomia in patients with diabetes, and the absence of patients with Chagas disease, which may limit generalization of the results to more diverse populations. The cross-sectional nature of the study precludes longitudinal assessment of patients and, consequently, analysis of outcomes.

## Conclusion

In our study, HR assessment with resting ECG in patients with HFrEF and sinus rhythm proved to be an accurate and reliable method, corroborating its widespread use in clinical practice. However, agreement was suboptimal in one third of patients with HR below 70 bpm on ECG; thus, 24-hour Holter monitoring should be considered in this situation.
